# Supplementary material for: Multi‐dataset identification of innovative feature genes and molecular mechanisms in keratoconus
Source: J Cell Mol Med. 2024 Sep 19;28(18):e70079. doi: 10.1111/jcmm.70079 (PMC11412914; doi:10.1111/jcmm.70079)
Supplement: Supplementary file 2 — Material S2. [file JCMM-28-e70079-s002.zip › 7.GSEA analysis/2.ATOH7/KEGG_VASCULAR_SMOOTH_MUSCLE_CONTRACTION.html]

Details for gene set KEGG\_VASCULAR\_SMOOTH\_MUSCLE\_CONTRACTION[GSEA]

|  || Dataset | ATOH7.group.cls#h\_versus\_l |
| Phenotype | group.cls#h\_versus\_l |
| Upregulated in class | h |
| GeneSet | KEGG\_VASCULAR\_SMOOTH\_MUSCLE\_CONTRACTION |
| Enrichment Score (ES) | 0.3738567 |
| Normalized Enrichment Score (NES) | 1.3505793 |
| Nominal p-value | 0.075510204 |
| FDR q-value | 0.3498147 |
| FWER p-Value | 0.933 |
Table: GSEA Results Summary

  

Fig 1: Enrichment plot: KEGG\_VASCULAR\_SMOOTH\_MUSCLE\_CONTRACTION      
 Profile of the Running ES Score & Positions of GeneSet Members on the Rank Ordered List

  

| SYMBOL | TITLE | RANK IN GENE LIST | RANK METRIC SCORE | RUNNING ES | CORE ENRICHMENT || 1 | KCNMB4 | na | 135 | 0.738 | 0.0462 | Yes |
| 2 | PRKACB | na | 829 | 0.474 | 0.0584 | Yes |
| 3 | PRKCE | na | 1002 | 0.447 | 0.0838 | Yes |
| 4 | RAMP1 | na | 1244 | 0.415 | 0.1050 | Yes |
| 5 | KCNMB2 | na | 1560 | 0.381 | 0.1218 | Yes |
| 6 | PLA2G1B | na | 1671 | 0.371 | 0.1438 | Yes |
| 7 | PLCB1 | na | 1718 | 0.367 | 0.1674 | Yes |
| 8 | PRKACG | na | 1784 | 0.361 | 0.1900 | Yes |
| 9 | PLA2G2C | na | 2003 | 0.344 | 0.2071 | Yes |
| 10 | ADRA1B | na | 2080 | 0.338 | 0.2278 | Yes |
| 11 | CYP4A22 | na | 2223 | 0.328 | 0.2460 | Yes |
| 12 | PRKCB | na | 2281 | 0.324 | 0.2664 | Yes |
| 13 | EDNRA | na | 2486 | 0.310 | 0.2815 | Yes |
| 14 | PLA2G2A | na | 2876 | 0.287 | 0.2898 | Yes |
| 15 | ACTG2 | na | 3324 | 0.264 | 0.2948 | Yes |
| 16 | CALML6 | na | 3497 | 0.256 | 0.3072 | Yes |
| 17 | ADORA2B | na | 3930 | 0.239 | 0.3110 | Yes |
| 18 | AVPR1A | na | 4160 | 0.231 | 0.3200 | Yes |
| 19 | MAP2K1 | na | 4240 | 0.228 | 0.3332 | Yes |
| 20 | CALCRL | na | 4461 | 0.219 | 0.3417 | Yes |
| 21 | AGTR1 | na | 4501 | 0.218 | 0.3554 | Yes |
| 22 | ARHGEF1 | na | 4976 | 0.202 | 0.3555 | Yes |
| 23 | ADCY3 | na | 5082 | 0.198 | 0.3659 | Yes |
| 24 | MYLK2 | na | 5529 | 0.184 | 0.3655 | Yes |
| 25 | MYL9 | na | 5666 | 0.180 | 0.3739 | Yes |
| 26 | ADORA2A | na | 6741 | 0.150 | 0.3530 | No |
| 27 | MYLK3 | na | 7050 | 0.142 | 0.3538 | No |
| 28 | ITPR1 | na | 7473 | 0.131 | 0.3505 | No |
| 29 | PRKCA | na | 7938 | 0.120 | 0.3452 | No |
| 30 | GNA13 | na | 8568 | 0.107 | 0.3343 | No |
| 31 | CALD1 | na | 8691 | 0.105 | 0.3379 | No |
| 32 | CACNA1S | na | 8751 | 0.103 | 0.3432 | No |
| 33 | ARHGEF12 | na | 8908 | 0.101 | 0.3455 | No |
| 34 | NPR2 | na | 9313 | 0.093 | 0.3402 | No |
| 35 | CALM3 | na | 10070 | 0.080 | 0.3238 | No |
| 36 | NPR1 | na | 10142 | 0.079 | 0.3271 | No |
| 37 | ARAF | na | 10150 | 0.079 | 0.3323 | No |
| 38 | ADCY8 | na | 10448 | 0.075 | 0.3287 | No |
| 39 | ARHGEF11 | na | 10528 | 0.073 | 0.3314 | No |
| 40 | ADRA1A | na | 11372 | 0.061 | 0.3112 | No |
| 41 | PRKACA | na | 12347 | 0.048 | 0.2863 | No |
| 42 | PRKX | na | 12697 | 0.044 | 0.2792 | No |
| 43 | GNA12 | na | 13021 | 0.040 | 0.2726 | No |
| 44 | PPP1CC | na | 14047 | 0.030 | 0.2450 | No |
| 45 | PRKCH | na | 14323 | 0.027 | 0.2390 | No |
| 46 | RAMP3 | na | 14494 | 0.026 | 0.2358 | No |
| 47 | MAPK1 | na | 14536 | 0.025 | 0.2363 | No |
| 48 | GNAS | na | 14632 | 0.024 | 0.2352 | No |
| 49 | PTGIR | na | 14773 | 0.023 | 0.2328 | No |
| 50 | RAF1 | na | 15219 | 0.019 | 0.2212 | No |
| 51 | PLA2G4A | na | 15227 | 0.019 | 0.2223 | No |
| 52 | PPP1R12A | na | 15265 | 0.019 | 0.2225 | No |
| 53 | RHOA | na | 15382 | 0.018 | 0.2204 | No |
| 54 | PLA2G6 | na | 15813 | 0.014 | 0.2089 | No |
| 55 | MYL6 | na | 15872 | 0.014 | 0.2082 | No |
| 56 | ACTA2 | na | 16142 | 0.012 | 0.2012 | No |
| 57 | CALM1 | na | 16251 | 0.011 | 0.1988 | No |
| 58 | MYH11 | na | 16745 | 0.007 | 0.1851 | No |
| 59 | MAPK3 | na | 16821 | 0.006 | 0.1833 | No |
| 60 | PPP1CA | na | 17263 | 0.002 | 0.1707 | No |
| 61 | PRKCD | na | 17924 | -0.003 | 0.1519 | No |
| 62 | MYL6B | na | 17946 | -0.003 | 0.1515 | No |
| 63 | PLA2G12A | na | 18221 | -0.006 | 0.1440 | No |
| 64 | GNA11 | na | 18372 | -0.007 | 0.1402 | No |
| 65 | PLA2G2E | na | 18403 | -0.008 | 0.1398 | No |
| 66 | ITPR3 | na | 18764 | -0.011 | 0.1301 | No |
| 67 | KCNMB3 | na | 18927 | -0.012 | 0.1263 | No |
| 68 | ROCK2 | na | 19403 | -0.016 | 0.1137 | No |
| 69 | PPP1CB | na | 19627 | -0.018 | 0.1085 | No |
| 70 | PRKG1 | na | 20059 | -0.022 | 0.0975 | No |
| 71 | CALML3 | na | 20596 | -0.028 | 0.0839 | No |
| 72 | GNAQ | na | 20613 | -0.028 | 0.0854 | No |
| 73 | MAP2K2 | na | 20653 | -0.028 | 0.0861 | No |
| 74 | ADCY9 | na | 20701 | -0.029 | 0.0867 | No |
| 75 | PLCB4 | na | 20753 | -0.029 | 0.0872 | No |
| 76 | CYP4A11 | na | 21497 | -0.037 | 0.0683 | No |
| 77 | ADCY2 | na | 21650 | -0.039 | 0.0665 | No |
| 78 | KCNMB1 | na | 21679 | -0.039 | 0.0684 | No |
| 79 | PPP1R14A | na | 21785 | -0.041 | 0.0681 | No |
| 80 | CALM2 | na | 21949 | -0.042 | 0.0663 | No |
| 81 | CACNA1C | na | 22079 | -0.044 | 0.0655 | No |
| 82 | RAMP2 | na | 22631 | -0.050 | 0.0530 | No |
| 83 | MYLK | na | 22661 | -0.050 | 0.0556 | No |
| 84 | JMJD7-PLA2G4B | na | 23573 | -0.063 | 0.0336 | No |
| 85 | PRKCQ | na | 23761 | -0.066 | 0.0327 | No |
| 86 | PLCB3 | na | 24121 | -0.071 | 0.0271 | No |
| 87 | PLA2G3 | na | 24134 | -0.071 | 0.0316 | No |
| 88 | KCNMA1 | na | 24776 | -0.082 | 0.0187 | No |
| 89 | BRAF | na | 25048 | -0.086 | 0.0167 | No |
| 90 | ADCY5 | na | 25230 | -0.089 | 0.0175 | No |
| 91 | ADCY6 | na | 25322 | -0.091 | 0.0211 | No |
| 92 | ROCK1 | na | 25698 | -0.098 | 0.0169 | No |
| 93 | PLA2G5 | na | 25954 | -0.103 | 0.0165 | No |
| 94 | CACNA1D | na | 26374 | -0.112 | 0.0121 | No |
| 95 | ADCY7 | na | 26708 | -0.119 | 0.0105 | No |
| 96 | ITPR2 | na | 26843 | -0.122 | 0.0149 | No |
| 97 | PLA2G10 | na | 27060 | -0.127 | 0.0173 | No |
| 98 | CACNA1F | na | 27070 | -0.127 | 0.0257 | No |
| 99 | PPP1R12B | na | 27096 | -0.128 | 0.0336 | No |
| 100 | PLA2G4E | na | 27235 | -0.132 | 0.0386 | No |
| 101 | ADCY4 | na | 27391 | -0.136 | 0.0433 | No |
| 102 | PLA2G2F | na | 27530 | -0.139 | 0.0488 | No |
| 103 | PLCB2 | na | 28336 | -0.160 | 0.0364 | No |
| 104 | PLA2G2D | na | 28487 | -0.164 | 0.0432 | No |
| 105 | AVPR1B | na | 28730 | -0.171 | 0.0478 | No |
| 106 | PRKCG | na | 28978 | -0.178 | 0.0528 | No |
| 107 | CALML5 | na | 29931 | -0.208 | 0.0394 | No |
| 108 | ADCY1 | na | 30908 | -0.244 | 0.0278 | No |
| 109 | ADRA1D | na | 32355 | -0.317 | 0.0075 | No |
| 110 | PLA2G12B | na | 33562 | -0.422 | 0.0013 | No |
| 111 | GUCY1A2 | na | 33901 | -0.477 | 0.0239 | No |
Table: GSEA details [plain text format]

  

Fig 2: KEGG\_VASCULAR\_SMOOTH\_MUSCLE\_CONTRACTION      
 Blue-Pink O' Gram in the Space of the Analyzed GeneSet

  

Fig 3: KEGG\_VASCULAR\_SMOOTH\_MUSCLE\_CONTRACTION: Random ES distribution      
 Gene set null distribution of ES for **KEGG\_VASCULAR\_SMOOTH\_MUSCLE\_CONTRACTION**
